# Refining anesthesia practice – regional techniques, risk stratification, and evidence-based analgesia

**DOI:** 10.3325/cmj.2025.66.183

**Published:** 2025-06

**Authors:** Viktor Duzel, Miroslav Župčić, Tatjana Šimurina, Sandra Graf Župčić

**Affiliations:** 1 Northern Hospital Epping, Melbourne, Victoria, Australia; *viktor.duzel@nh.org.au*; 2 Department of Anesthesiology and Intensive Care Medicine, Rijeka University Hospital Center, Rijeka, Croatia; 3 Department of Clinical Sciences II, Faculty of Health Studies, University of Rijeka, Rijeka, Croatia; 4 Department of Anesthesiology, Resuscitation and Intensive Care Medicine, Zadar General Hospital, Zadar, Croatia; 5 Faculty of Medicine, Josip Juraj Strossmayer University of Osijek, Osijek, Croatia; 6 Department of Health Studies, University of Zadar, Zadar, Croatia; 7 Department of Neurology, Rijeka University Hospital Center, Rijeka, Croatia; 8 Faculty of Biotechnology and Drug Development, University of Rijeka, Rijeka, Croatia; Duzel et al: Refining anesthesia practice

This issue of the *Croatian Medical Journal* presents a selection of original articles, essays, and a case report that reflect a progressive shift in anesthetic practice – away from rigid protocols toward patient-specific, physiology-informed decision-making. The common thread across these studies is a careful adaptation of anesthetic technique to surgical burden, comorbidity profile, and evolving evidence. These contributions collectively signal a growing emphasis on regional anesthesia, rational opioid use, and perioperative risk stratification in complex surgical patients. Here, we present several highlights of the issue content. Eljuga et al (1) describe an opioid-free protocol for mastectomy with immediate reconstruction using a combination of thoracic paravertebral (TPVB), pectoral (PECS I), and serratus anterior plane (SAP) blocks. Intraoperative opioid avoidance was successful; however, postoperative opioid use remained comparable to standard care. Despite this, pain scores remained low for up to 10 days postoperatively, which suggests that regional blockade provided durable analgesia. These results support multimodal regional strategies, though they also underscore that opioid-free intraoperative care does not guarantee complete postoperative opioid sparing. This observation is reinforced by the essay by Kukić et al (2), who discussed differences between TPVB, PECS II, and SAP blocks for breast-conserving surgery. The literature suggests that TPVB consistently provides superior analgesia and opioid reduction but is technically demanding. PECS II and SAP blocks offer safer, easier alternatives, but demonstrate variable efficacy, especially when used in isolation (3). The authors advocate tailoring block selection to surgical extent, anticipated pain intensity, and institutional experience, aligning with the emerging consensus that fascial plane blocks should be viewed as complements rather than substitutes for deeper paravertebral blockade (2). Gačo Rački et al (4) comment on the evidence base for neuraxial morphine and conclude that, when feasible, intrathecal administration offers superior analgesia with lower systemic exposure compared with epidural delivery (5-9). Concerns related to pain management are tackled by Milić et al (10), who retrospectively assessed analgesics use in a large cohort of patients treated at the Emergency Department of Krapina-Zagorje County. This single-center study shows there is still a lot of room for improvement when it comes to treating pain adequately by implementing up-to-date pain management protocols and ensuring continuous educational approaches.

All these observations contribute meaningfully to enhanced recovery after surgery protocols and advocate for evidence-based refinement of analgesic guidelines. However, the known risks of delayed respiratory depression and unpredictable duration warrant caution and appropriate monitoring. Višković Filipčić et al (11) present a single-center retrospective study of major cardiovascular events during orthotopic liver transplantation. The incidence of perioperative events approached 9%, with older age, metabolic liver disease, donor hypertension, and prolonged cold ischemia identified as key risk factors. Given the increasing age and comorbidity of transplant candidates, the occurrence of severe adverse cardiovascular events was markedly higher than previously documented. The authors propose the use of scoring systems including known risk variables to identify patients at risk of adverse cardiovascular events; hence improving surveillance for early identification and intervention as supported by international transplant societies (12). A retrospective study by Krecak et al (13) on 1196 patients undergoing elective non-cardiac surgeries investigated whether preoperative polycythemia may be associated with inferior postoperative outcomes. The authors used the World Health Organization criteria to stratify patients into three categories according to their preoperative hemoglobin values: anemic, normal, and polycythemic. Their primary aim was to determine a 30-day postoperative composite outcome consisting of death, thrombosis, bleeding, and the need for postoperative red blood cell transfusion. Interestingly, the composite outcome was most frequent in patients with preoperative polycythemia, predominantly due to the highest frequency of postoperative bleeding events. Moreover, the association of polycythemia with the composite outcome was independent of patient sex, surgery risk, comorbidities, American Society of Anesthesiologists score, and the use of antithrombotic medications. Although anesthesiologists are usually more afraid of preoperative anemia, this interesting study suggests that patients presenting with preoperative polycythemia require careful perioperative surveillance. Župčić et al (14) describe the use of unilateral TPVB and remimazolam sedation for open cholecystectomy in a patient with severe cardiac disease and an implantable cardioverter defibrillator. Despite avoiding general anesthesia and neuraxial blockade, the team achieved adequate surgical anesthesia and maintained hemodynamic stability throughout the procedure. This case illustrates the flexibility and value of regional techniques in patients for whom standard approaches carry elevated risk. Tomulić Brusich et al (15) discuss the use of ultrasound-guided regional anesthesia (UGRA) in the intensive care setting. UGRA is associated with reduced opioid use, improved pain control, and fewer sedative complications in mechanically ventilated patients; yet it remains underutilized. The authors attribute this gap to limited clinician training and insufficient institutional protocols, joining growing calls for expanded UGRA capability in critical care environments. Tarčuković et al (16) explore the use of a honey-based oral carbohydrate solution as a natural alternative to commercial preparations in elective laparoscopic cholecystectomy. Their randomized study reveals promising benefits in terms of improved gastric motility and reduced postoperative symptoms such as thirst and pain. However, the honey solution was also associated with elevated cortisol and interleukin 6 levels, which suggests a stronger stress response. These findings highlight both the potential and the complexity of incorporating natural carbohydrate sources into preoperative protocols. As an extended interest in this issue, Miklić Bublić et al (17) reflect on transcutaneous vagal nerve stimulation as a new technique that may induce neuromodulation in brain trauma patients, thus alleviating the damage and enhancing recovery by reducing epileptic seizures and brain edema, as well as potentially improving brain tissue oxygenation and reperfusion of the penumbra.

Taken together, the studies published in this issue reflect a deliberate shift toward more precise, risk-adapted anesthetic care. Regional techniques are being re-evaluated not only as adjuncts but as primary anesthetic strategies in appropriately selected patients. Morphine administration is being refined to balance efficacy and safety. Meanwhile, perioperative risk factors such as cardiac vulnerability and erythrocytosis are gaining overdue attention. These contributions point toward a more patient-centered, evidence-based future in anesthesiology.

## References

[1] EljugaD MužarRM JurišićI PeršecJ EljugaK JamanJ Regional anesthesia without opioid administration in mastectomy surgeries followed by breast reconstruction with implants: a randomized controlled trial. Croat Med J 2025 66 213 9 40635342

[2] KukićL ŽupčićM Višković FilipčićN SulenN DuzelV TonkovićD Is paravertebral block the optimal analgesic modality for non-mastectomy breast surgery? Croat Med J 2025 66 238 40 40635347

[3] Gabriel RA Curran BP Swisher MW Sztain JF Tsuda PS Said ET Paravertebral versus pectoralis-II (interpectoral and pectoserratus) nerve blocks for postoperative analgesia after nonmastectomy breast surgery: A randomized, controlled, observer-masked noninferiority trial. Anesthesiology 2024 141 1039 50 10.1097/ALN.0000000000005207 39186671

[4] Gačo RačkiN ŽupčićM Graf ŽupčićS RačkiV ŠimurinaT DuzelV Is there a difference in the effectiveness of neuraxial morphine administration? Croat Med J 2025 66 235 7 40635346

[5] Koning MV Teunissen AJW van der Harst E Ruijgrok EJ Stolker RJ Intrathecal morphine for laparoscopic segmental colonic resection as part of an enhanced recovery protocol: A randomized controlled trial. Reg Anesth Pain Med 2018 43 166 73 29219935 10.1097/AAP.0000000000000703 PMC5794252

[6] Lokin JLC, Savelkoul C, van Eekeren RRJP, et al. Patients' perception of the duration of analgesia provided by intrathecal bupivacaine/morphine after laparoscopic colorectal surgery: a prospective cohort study. APS. 2024;2(19).

[7] El-Mallah JC Greene AC Clegg TJ Shah SJ El-Mallah SN Vining CC Comparison of epidural infusion versus intrathecal morphine block as part of enhanced recovery after open pancreatoduodenectomy. J Surg Oncol 2024 129 869 75 10.1002/jso.27574 38185838

[8] Boisen ML McQuaid AJ Esper SA Holder-Murray J Zureikat AH Hogg ME Intrathecal morphine versus nerve blocks in an enhanced recovery pathway for pancreatic surgery. J Surg Res 2019 244 15 22 10.1016/j.jss.2019.05.049 31279259

[9] Pirie KP Wang A Yu J Teng B Doane MA Myles PS Postoperative analgesia for upper gastrointestinal surgery: a retrospective cohort analysis. Perioper Med (Lond) 2023 12 40 10.1186/s13741-023-00324-0 37464387 PMC10355044

[10] MilićM Barić GrgurevićA VrdoljakJ FučkarK BrundulaA Inadequate pain management in prehospital emergency care: a retrospective study from Krapina-Zagorje County. Croat Med J 2025 66 220 6 40635343

[11] Višković FilipčićN Tomulić BrusichK MiloševićM PerkovS ŽupčićM Filipec KanižajT Predictors of perioperative cardiac events in liver transplantation: a retrospective study. Croat Med J 2025 66 194 203 40635340

[12] De Luca L Kalafateli M Bianchi S Alasaker N Buzzetti E Rodríguez-Perálvarez M Cardiovascular morbidity and mortality is increased post-liver transplantation even in recipients with no pre-existing risk factors. Liver Int 2019 39 1557 65 10.1111/liv.14185 31233663

[13] KrečakI Valovičić KrečakM Preoperative polycythemia may be associated with inferior postoperative outcomes. Croat Med J 2025 66 186 93 40635339

[14] ŽupčićM Tomulić BrusichK NadarevićT Graf ŽupčićS ĐuzelV RedžepiG The application of paravertebral block for open cholecystectomy in high-risk patient with implantable cardioverter-defibrillator: a case report. Croat Med J 2025 66 227 30 40635344

[15] Tomulić BrusichK DubrovićM ŽupčićM Why we should insist on education and implementation of ultrasound-guided regional anesthesia in the intensive care units. Croat Med J 2025 66 231 4 40635345

[16] Tarčuković J Klarica L Dugonjić M Vuksan I Pavlešić T Šonjić P The effects of honey solution on postoperative stress, gastric motility, and patient comfort: a randomized controlled trial. Croat Med J 2025 66 204 12 40635341 10.3325/cmj.2025.66.204 PMC12426867

[17] Miklić BublićM TonkovićD Vrbanović MijatovićV A case for a bigger role of transcutaneous vagus nerves stimulation in brain injury rehabilitation. Croat Med J 2025 66 241 2 40635348

